# Monthly Summary

**Published:** 1882-02

**Authors:** 


### MONTHLY SUMMARY.

---

*Uniting Gold to Amalgam in Tooth Filling.*—I would like to refer to a use I have been making of amalgam for several years, and which I reported to the State society some four or five years ago. I advocated at that time that in many instances of large cavities on approximal surfaces,—of bicuspids and molars particularly—they should be partly filled with amalgam, to be followed at the same sitting with gold, carrying out the filling to the grinding-surface of the tooth, if one desired to avoid a full amalgam filling. Without going into a lengthy discussion of the arguments in favor, or the objections that might be offered, let me say that I have seen a great many of those fillings, and have watched them with much solicitude and anxiety, but I have never seen a single instance where there has been any recurrence of decay at the cervical edge of the cavity. In every case that I have seen I have found the amalgam discolored on the surface, but the gold as bright as any gold filling and the tooth preserved from further decay. I have been querying, why this result; whether it was attributable entirely to the fact that with the amalgam I got perhaps a closer adaptation to the cervical edge of the cavity than I would with gold, or whether it was owing to some galvanic condition of the different metals in contact, or what? But this I do know, that sooner or later the majority of operations, under like circumstances, when made with gold, are giving out at that portion of the cavity, no matter by whom they are made. I say a majority, not because I have had an opportunity of examining all the fillings made by every operator, but because I am constantly seeing the work of the most skilful operators in gold giving out at the point referred to within a few years. There is not a dentist who is not seeing this almost daily, and among his own patients, if he would but have the candor to admit it. I have been puzzled for an explanation of the success where amalgam has been used in this manner. There is no evidence of shrinkage of the amalgam that is placed in the upper part of the cavity. Whatever amalgam may do under other circumstances, that amalgam shows no shrinkage after it is put in. It remains perfectly tight, and the tooth does not discolor in contact with it. In filling a tooth in this way the amalgam occupies a third or a half of the cavity, and the gold is immediately forced into it. For some little time the gold will absorb the mercury and take up all the excess. After that ceases, the gold shows its true gold appearance, and unites or welds in the usual way. The filling can be finished as soon as the gold is packed. The amalgam will be found sufficiently hard to be finished up to the cervical edge. We see here two results; one is that the gold has taken up all the excess of the mercury that can possibly be taken from the mass of amalgam, preventing the possibility of shrinkage ; and, secondly, such a filling is in a better condition to make a perfect flush edge at the cervical border. There is little or no risk of the difficulty so often found with gold fillings, of the filling standing out and forming a lodgment for foreign matters, which will end in decay, because the amalgam has not become so hard but that it can be easily brought flush with the body of the tooth. This may be the explanation of the results that I believe will almost invariably attend such operations. I spoke of this before the State society a few years since with some timidity, for the reason that there is such a prejudice in the minds of almost every one against amalgam—and especially as the charge is often made that amalgam is only used by those who cannot put in a gold filling. It was not for the reason that gold could not be used but because I saw gold fillings were constantly giving out, that I felt obliged to resort to something for the good of the patient, and the result has been so successful and uniform that I felt it a duty to give somebody else the benegt of it.—*Dr. Kingsly in Trans. of Odontological Society.*

---

*The Use of Platinum in Amalgam Alloys.*—It has been for some years a disputed point as to whether platinum is or is not inert sn amalgams. Many who are quite competent to judge fairly, declare that it is totally useless, others equally competent state that it is a necesiity in a really good amalgam. Until recently, knowing the great difficulty of getting platinum properly in combination, I have been led to believe that the question rested solely on the point as to whether the platinum existed as a mechanical mixture or, as it should be, in chemical combination, a state in which it certainly can be obtained by proper management.

Having given a sample which I know contained the disputed metal in combination, to one of its opponents, he still declared that the same alloy was better without the platinum than with it. Fortunately, he sent me the test plugs he had mixed, and an analysis of these showed that both had been mixed with a very large excess of mercury. On repeating his tests under his conditions I obtained the same result, that the platinum, if anything made the alloy worse, and I fortunately also got at the explanation. It would appear that platinum, like palladium, combines rapidly and strongly with the mercury, and that its power of setting lasts for a very short time. If a great excess of mercury is used, sufficient to make the whole mass set slowly, there is for the first few minutes a certain point, the platinum having exerted its settling power and became inert. If the mass is now disturbed and worked up, the platinum in combination gets apparently broken up, and have no further powerof setting, it simply exists as an inert foreign mixture, and the result is really better without the platinum than with. It would appear, therefore, that this metal is useful only in alloys which are so made and mixed as to set quickly, and that their ultimate hardness is entirely dependent on the setting by first intention being undisturbed; an alloy containing platinum must therefore not only be quickly worked, but it must be made and worked in such a manner that it is not liable to be disturbed until hard. A similar destruction of setting power may be noticed in Portland cement, which if disturbed when partially hard never forms a solid mass, its "growing" together is stopped and the result is a brittle mass with little if any coherence; the same mixing if left undisturbed forming a hard stone which is broken or cut only with great difficulty.—*Brit. Jour. of Dental Science.*

---

*Catching Cold—Remedies.*—While it is easy to take cold in midsummer, colds are usually more prevalent when low temperature prevails, though less in clear, steady winter than during the variable spring and autumn. "Catching cold," is usually the result of inequality of temperature in two parts of the body, especially adjacent parts, which disturbs the uniform circulation of the blood. At the place where this disturbance occurcs, "congestion" arises, that is, a rush of blood to a part from one direction faster than it is carried off by the chilled blood vessels in the other direction, and this produces serious results if not speedily remedied. This diseased condition may extend over the whole body, affecting most severely any organ already weak.

Thus a cold may come from damp or chilled feet; from even a slight draft of air blowing through a crack, upon one side or portion of the body and cooling it; from standlng near a fire or stove, and heating one side while the other remains comparatively cold; from warmer clothing on one part of the body than on another; from lightly dressing the arms and lower limbs, or leaving them naked; from standing over a hot register; from the chilling evaporation of water or moisture, from a portion only of one's clothing—; in general, from any cause producing inequality of temperature.

The causes of a cold, named, indicate how to avoid one. Maintaining general vigor by nourishing, well digestsd food, gives one power to resist an attack. When to be especially exposed, a little tonic, as a grain or two of quinine, taken in advance, may be useful. Stimulants, like alcoholic liquors, are but a temporary aid; the reaction after the first stimulating effects, leaves one more subject to take cold than if the stimulant had been omitted.

Simple remedies will usually remove a cold, if taken promptly, before the congestion has produced serious disorganization. When struck with a sense of chillnsss, 15 to 30 drops of Aromatic Spirits of Ammonia, in half a tumbler of water, will often start a uniform circulation all through the body, as this quickly enters the whole blood and is stimulating. Soaking the feet in warm water, gradually adding warmer water as long as it can be borne, draws off the blood from all the rest of the body, and often relieves congestion in any local part. Smart friction upon any part or the whole of the skin surface, or a uniform surface sweating, produces like results. But in these cases, special care must be taken to prevent after-chilling of the feet, or any rther part. After the feet heating, wipe dry quickiy and cover them warmly.

The best remedy we have found for a recent cold is a moderate movement of the bowels with castor oil, or caustic or other mild cathartic magnesia. This produces a flow of fluid, drawn from the blood to the alimentary canal, and thus reduces the pressure upon any one congested point, just as drawing off part of the water from a flooded pond relieves pressure upon a weakened dam or embankment. This is to be followed by keeping the body warm and comfortable, and toning it up with good food, or a simple tonic like quinine, "Feeding a cold," prior to taking a cathartic, is the worst possible treatment It is only adding material to increase the congestion.—*American Agriculturist.*

---

*Oxygenation of Amalgam.*—Dr. G. A. Mills says: "I was reading the *Missouri Dental Journal* last night, and noticed some remarks by Dr. S. B Palmer, of Syracuse, who holds the idea that there is danger coming from what we term the standard amalgam. He claims that by raising the quality to prevent tarnishing, we are destroying one of the qualities which, to his mind, is important to the amalgam. He says the oxydation is what we want. By improving the quality we lose this; and he says that, in his judgment, the new standards that are coming out are going to disappoint a great many men. It is a query in my mind if that can be so. I will refer to a case that will please Dr. Clowes. There were two small fillings—one in a lower cuspid and one in the upper bicuspid—in the mouth of a servant girl. I took out the filling from the lower tooth, and found the pulp dead, and noticed that the tooth was very much softened under the filling. I thought nothing of it then. She also complained of the upper tooth; and I said, if it does not become quiet I will see to it. The next week she came in again, and, on taking out the small approximal filling, I found the structure of the tooth was destroyed to such an extent that it was a black, gray, and yellow mass, and the pulp was exposed. I took it to be cadmium amalgam, against which Dr. Clowes has so often warned us. I spoke to some gentlemen of this case, and they said at once, 'Boston amalgam!'"

---

*Tempering Steel*—According to a Sheffield paper a very fine preparation for making very hard steel is composed of wheat flour, salt and water, using, say, two teaspoonfuls of water, one-half a teaspoonful of flour and one of salt. Heat the steel to be hardened enough to coat it with the paste by immersing it in the composition, after which heat it to a cherry red and plunge it into soft water. If properly done the steel will come out with a beautiful white surface. It is said that Stubs' dies are hardened in this manner.

